# The New York Institute of Stomatology

**Published:** 1905-10

**Authors:** 

**Affiliations:** The New York Institute of Stomatology


### Reports of Society Meetings.

### THE NEW YORK INSTITUTE OF STOMATOLOGY.

A MEETING of the Institute was held at the Chelsea, 222 West Twenty-third Street, New York, on Tuesday evening, June 6, 1905, the President, Dr. C. O. Kimball, in the chair.

The minutes of the last meeting were read and approved.

### COMMUNICATIONS ON THEORY AND PRACTICE.

*Dr. H. W. Gillett.*—I wish to call attention to the little paper points put up by Johnson & Johnson for drying out pulp-canals. At my request they have made some a little shorter and thicker than their regular stock. I find these a very great convenience. It may be a convenience to members to know that they can get these special points by ordering a quantity.

*Dr. F. Milton Smith.*—I wish to offer a suggestion in regard to a method of evacuating pus from a cavity. The case where I had occasion to use the method was a right lower bicuspid tooth. I mentioned it to Dr. Bogue, and he said that he had never heard of it before. It is sometimes difficult, after the opening is made through the root of a lower tooth, to get the pus to come up through it. To facilitate this I took a little Dunn syringe and placed the point into the canal. After squeezing the bulb together and holding it there, I packed the space around the point with gutta-percha. Then releasing the bulb, I found the suction sufficient to bring up the pus.

Dr. Sinclair Tousey presented a little appliance to be used in connection with X-ray work in making skiagraphs of the jaw. It consisted of a film-holder to which was attached an indicator so arranged that it would facilitate the adjusting of the apparatus for any particular field in and about the mouth. Negatives made in this way were more quickly done, but were not quite so sharp. Dr. Tousey exhibited the apparatus together with some of the negatives.

Dr. Leo Green mentioned a case of fracture of the inferior maxilla which was readily reduced by means of an aluminum cap splint. This did away with the necessity of feeding through a tube. He thought with a little more attention to details the interdental splint might be discarded.

Dr. S. E. Davenport read a paper entitled "Some Partial Impressions."

(For Dr. Davenport's paper, see page 645.)

DISCUSSION.

*Dr. Strang.*—I can indorse Dr. Davenport's method of taking a preliminary impression and making therefrom a cup. I frequently have had occasion to take my first impression without regard to the teeth, simply vulcanizing on that model the base on which the teeth are to be articulated, and with that in the mouth getting the correct articulation. I remember about ten years ago I had a very perplexing case. The difficulties of getting a good impression of that mouth seemed insurmountable. I made the plate upon which the teeth were finally mounted in two sections. I took the impression of one side of the mouth first and vulcanized this half, and then got my correct impression of the other side and vulcanized that. Placing them together, I had a correct plate on which to articulate the teeth. The result was very satisfactory.

*Dr. F. L. Fossume.*—In difficult cases where undercuts exist, I use smooth, nickel-plated impression cups, which are oiled, and after the plaster is hardened in the mouth the impression cup is slipped off and the plaster is split with a knife and removed in sections, and these are again placed back in the cup.

Where a metal plate is to be struck up on such models, the teeth forming the undercut should be sawed half through and then broken off, and after the metal die is obtained and the plate swaged the teeth are carefully placed back on the model and waxed in position with hard wax. In this way the whole denture with clasps and lugs can be completed on one model, which is of great importance, as it avoids taking another impression.

Dr. C. W. Strang, of Bridgeport, Conn., read a paper entitled "Errors, Mistakes, and Failures, Old and New."

(For Dr. Strang's paper, see 639.)

DISCUSSION.

*The President.*—I had the other day a personal illustration bearing out some of the points in Dr. Strang's paper. My second son, an interne in one of the New York hospitals, while fooling with one of his associates, acquired a twisted fracture of one of the metacarpal bones. He came down with his wrist all done up in the proper manner. He told me that he had learned more in twenty-four hours regarding such fractures than he had learned during his entire medical course. He proceeded to enumerate the things he had found out from this personal experience that he could not have gotten from his teachers.

I am sure that any of us who, in the course of our experience, have required the manipulation in our own mouths of some other practitioners, have learned more than we had learned in long years of ordinary practice. It has been said that no one ought to practise dentistry who did not have a tooth to be filled at least three or four times a year, so that he might know something about the way in which he is treating his friends.

*Dr. E. A. Bogue.*—It has seemed to me that Dr. Strang's experience is the experience of us all. He has stated that at first he tried to follow the teachings of Dr. Atkinson, but after abandoning them he did better. I remember at one time having consulted thirty-two of my fellow-practitioners in regard to a certain point, finding eventually that every one of them was in the wrong. That is why I have my thousand models now for reference.

I saw to-day a case that seemed to me to be one of extreme error. It was one of ten or more inlays. They were of metal and on the grinding surfaces of the anterior teeth above and below. All of them had pins set into the pulp-chambers of the various teeth. The articulation was such that none of the upper incisors met the lower. These heavily armored teeth did not touch each other at all. Why these teeth should have been shod to that extent I do not know.

Regarding the case mentioned by Dr. Strang, I could not help wondering why he did not have a microscopical examination made.

*Dr. Strang.*—That was seven years ago, and the facilities for such an examination were not as available as now.

*Dr. F. Milton Smith.*—I am thankful, gentlemen, that I have not a monopoly on this subject. I am delighted to hear of Dr. Strang's mistakes. One or two of my own mistakes are still in my memory.

I have not in years gone by regulated sets of teeth as I should have done, and I am beginning to have patients come in to me who have been my patients for fifteen, twenty, twenty-five, or twenty-eight years, and I am beginning to realize my former neglect. This is one of the mistakes that I have made.

Another very serious mistake that I have made is that in order to make room in certain cases I have sacrificed an upper bicuspid and a lateral incisor, lower. I wish some of you gentlemen could see that mouth.

*Dr. Bogue.*—Another mistake. Last Friday a young lady was in my chair, and she wanted to know why her teeth were decaying between the molars, as she was very careful in cleansing them. I explained to her that the reason was that when she was a child I did not know enough to regulate her teeth properly. "If I were doing it to-day I should see to it that all the thirty-two teeth articulated as nature had intended them. If I had done this you would not have the trouble you are now having."

It is not more than three years since our president said to me, at a meeting of the Reform Club, "Why, the first thing we know, this fellow will be claiming that the articulation of the teeth has something to do with the deposition of tartar." Dr. Davenport, who was with us at the time, immediately spoke up, "Well, it does." And he was right.

The wisdom-teeth, situated as they are at the angle of the jaw, are the key of the other teeth. They are the braces against which the other teeth rest. They are the bumpers set up at the station house against which the trains are to hit. Remove the braces, and what happens? The other teeth begin to separate a little bit, food crowds in between, and then deposits of tartar will begin to be found, and you may be pretty sure decay will go on there sooner or later.

*Dr. Gillett.*—I often feel thankful that two cases of first molar extraction are out of my reach and that I see them no more. One of them I never saw after the extraction was done. A case that I do see is one in which I insisted on having the deciduous molars extracted because they remained some time after their normal time. I did this with the assurance that the bicuspids would soon be making their appearance. I see that patient occasionally, but those bicuspids have never appeared, and it is a pretty sorry set of teeth. Of course, we do not do those things since we have had the X-ray, and the younger men may avoid such mistakes.

*Dr. Strang.*—Referring to Dr. Bogue's contention that the wisdom-teeth should always be saved, suppose, for example, in a patient eighteen or nineteen years of age the wisdom-teeth erupt and almost immediately begin to break down, and this in a mouth where all the other twenty-eight teeth are in good condition. It has seemed to me that in these cases, where it seems hopeless to save the wisdom-teeth, it would be better to take them out and preserve the remaining teeth in a fairly sound condition. The wisdom-teeth are not always good bumpers, because sometimes they point the other way.

*Dr. Bogue.*—The normal articulation of the teeth at about eighteen years of age involves twenty-eight teeth. Later on it may require thirty-two, but when the articulation is normal these teeth are almost self-cleansing. They do not need a brush, and they do not need a dentist very often, and when they wear down they leave cusps.

*Dr. L. C. LeRoy.*—I have in mind a case where the upper wisdom-teeth seemed to be not merely unnecessary, but positively an injury to the mouth. Before the third molars erupted the arch was practically perfect, with the exception that the lateral incisors were apparently too wide for the size of the upper centrals. This disarranged the anterior arrangement a little. The point I wish to make is that the cuspids, bicuspids, and molars occluded perfectly. When the upper wisdom-teeth erupted they caused considerable crowding and overlapping of the lateral incisors and disarrangement of the occlusion until the eruption of the lower third molars.

Regarding the surfaces of molars that are so prone to decay, I have treated such surfaces with nitrate of silver previous to filling with phosphate. The advent of copper marks the advent of a new era in the treatment of these cases and many so-called "soft" teeth of children.

*Dr. Gillett.*—Dr. Strang speaks of the nearly perfect mouths in which there have appeared these very imperfect wisdom-teeth, the extraction of which he advocates. It would be very interesting if he would go over these mouths again as they come to him and ascertain if possible if any of the things spoken of by Dr. Bogue have occurred, and show us casts to demonstrate whether the extraction of the wisdom-teeth in these cases has resulted in harm. Those of us who have been in practice know that it often takes more than ten years to develop results in the mouth. We so often misunderstand each other because the same words do not always mean the same thing to speaker and hearer. Casts showing results would be of great value in discussing this point.

*Dr. Strang.*—I might say that this suggestion of Dr. Gillett's has been followed, and that I have as yet seen no ill effects from these extractions.

It was moved and carried unanimously that a vote of thanks be extended to Dr. Strang for his very helpful paper.

Adjourned.

FRED. L. BOGUE, M.D., D.D.S.,
*Editor The New York Institute of Stomatology.*

